# Lessons learnt: Undertaking rapid reviews on public health and social measures during a global pandemic

**DOI:** 10.1002/jrsm.1580

**Published:** 2022-07-31

**Authors:** Eva A. Rehfuess, Jacob B. Burns, Lisa M. Pfadenhauer, Shari Krishnaratne, Hannah Littlecott, Joerg J. Meerpohl, Ani Movsisyan

**Affiliations:** ^1^ Institute for Medical Information Processing, Biometry and Epidemiology Ludwig‐Maximilians‐Universität Munich Germany; ^2^ Pettenkofer School of Public Health Munich Germany; ^3^ Department of Disease Control, Faculty of Infectious and Tropical Diseases London School of Hygiene and Tropical Medicine London UK; ^4^ DECIPHer (Centre for Development, Evaluation, Complexity and Implementation in Public Health Improvement), School of Social Sciences Cardiff University Cardiff UK; ^5^ Institute for Evidence in Medicine, Medical Center & Faculty of Medicine University of Freiburg Freiburg Germany; ^6^ Cochrane Germany Cochrane Germany Foundation Freiburg Germany

**Keywords:** COVID‐19, modeling studies, pandemic, public health, rapid review, scoping review

## Abstract

Public health and social measures (PHSM) have been central to the COVID‐19 response. Consequently, there has been much pressure on decision‐makers to make evidence‐informed decisions and on researchers to synthesize the evidence regarding these measures. This article describes our experiences, responses and lessons learnt regarding key challenges when planning and conducting rapid reviews of PHSM during the COVID‐19 pandemic. Stakeholder consultations and scoping reviews to obtain an overview of the evidence inform the scope of reviews that are policy‐relevant and feasible. Multiple complementary reviews serve to examine the benefits and harms of PHSM across different populations and contexts. Conceiving reviews of effectiveness as adaptable living reviews helps to respond to evolving evidence needs and an expanding evidence base. An appropriately skilled review team and good planning, coordination and communication ensures smooth and rigorous processes and efficient use of resources. Scientific rigor, the practical implications of PHSM‐related complexity and likely time savings should be carefully weighed in deciding on methodological shortcuts. Making the best possible use of modeling studies represents a particular challenge, and methods should be carefully chosen, piloted and implemented. Our experience raises questions regarding the nature of rapid reviews and regarding how different types of evidence should be considered in making decisions about PHSM during a global pandemic. We highlight the need for readily available protocols for conducting studies on the effectiveness, unintended consequences and implementation of PHSM in a timely manner, as well as the need for rapid review standards tailored to “rapid” versus “emergency” mode reviewing.

**Highlights**

**What is already known**

- Rapid reviews have gained considerable importance during the COVID‐19 response. However, this has exposed methodological challenges in conducting such reviews in the context of an ongoing pandemic.

**What is new**

- We describe three broad pandemic circumstances and the associated challenges encountered during rapid reviewing of COVID‐19‐related public health and social measures.
- We suggest that there is a difference between “rapid” and “emergency” mode reviewing, and that rapid review standards may need to be tailored to these different modes.
- We reflect on lessons learnt for planning rapid reviews, highlighting the value of formal scoping, including stakeholder consultations and scoping reviews, of multiple complementary reviews to capture the benefits and harms of measures, and of adaptable living reviews.
- We also suggest lessons learnt for conducting rapid reviews, emphasizing review team composition and collaborative working arrangements, the difficult balance between rigor and time savings in light of complexity and the challenge of synthesizing modeling studies.

**Potential impact for *Research Synthesis Methods* readers outside the authors' field**

- COVID‐19 related public health and social measures are multi‐sectoral in nature with a range of implications for society at large. Many of the challenges encountered and lessons learnt in conducting rapid reviews of such measures are also likely to apply to other multi‐sectoral interventions.

## 1 INTRODUCTION

In March 2020, the World Health Organization (WHO) declared the severe acute respiratory syndrome coronavirus 2 (SARS‐CoV‐2), and the associated disease, COVID‐19, a pandemic.^1^ Since then, the disease has spread globally and, as of May 2022, has caused more than 522 million cases and 6.2 million deaths and leading to severe economic and social impacts worldwide.^2^ Transmission of the virus primarily occurs through the inhalation of airborne droplets and aerosols.^3^ Consequences of an infection range from having no or limited symptoms to severe illness, including acute respiratory distress, severe pneumonia, renal failure, and death.^4^

Governments around the world have implemented a variety of measures to contain the spread of SARS‐CoV‐2 and minimize the impact of COVID‐19. COVID‐19‐related public health and social measures (COVID PHSM), also known as non‐pharmaceutical interventions, have played a central role and have been implemented nationally as well as in specific settings such as workplaces, childcare and educational settings and public transportation. They include lockdowns of varying intensity, full or partial border closures, full or partial school closures, as well as social distancing measures, mask wearing, and hygiene promotion,^5^ and heavily rely on compliance from the community, sometimes over prolonged periods of time, in order to be effective.^6^

While scientists have sought to assess the effectiveness of different COVID PHSM, multiple challenges have complicated these efforts. Due to the rapid onset and dynamic development of the pandemic, studies have largely relied on modeling to predict impact on transmission and health outcomes, especially during the first year of the pandemic.^7^ Further, studies have rarely assessed the influence of these measures on unintended, potentially negative, consequences, including health harms (both physical and psychosocial). In most countries, multiple measures have been implemented simultaneously to contain the spread of the virus, adding further difficulty to assessing the effectiveness of individual measures. Indeed, there has been very limited research on COVID PHSM as compared to biomedical treatments and vaccines, and the research that exists has many limitations.^8^, ^9^

Consequently, when undertaking evidence synthesis of COVID PHSM during the pandemic, we have experienced three broad pandemic circumstances and associated unique challenges. First, the *health problem has been evolving rapidly* and continues to evolve over time, due to changes in the virus (due to different variants), changes in the susceptibility of the host population (due to time‐varying recovery and immunization) and changes in context (e.g., perception and acceptance of risk, health system capabilities). In responding, evidence synthesis had to address and navigate the evolving questions and needs of decision‐makers (challenge 1). Second, the COVID‐19 pandemic has witnessed an *unprecedented level of research activity across disciplines and related rapid progression of the evidence base*. Initially, the evidence base in support of COVID PHSM was highly immature, with evidence synthesis efforts having to compromise on the types and quality of studies to be considered and resulting in very limited confidence in review findings. Over time, decisions had to be made regarding which types of evidence (e.g., modeling vs. empirical studies) and studies (e.g., different non‐randomized study designs and/or specific study features) could offer meaningful insights during a rapid expansion of the evidence base (challenge 2). Third, the pandemic has *increased decision‐makers'* “*appetite*” *and demand for evidence*, presenting an overall window of opportunity for science to influence decision‐making, as well as many small windows of opportunity for science to react to specific and time‐sensitive questions. In this context, evidence synthesis often had to produce complex reviews within a very short timescale of a few days and weeks (which we refer to as “emergency mode”) and, later on, a few months (which we refer to as “rapid mode”) (challenge 3). Evidence use in decision‐making may occur in different ways; instrumental (i.e., acting on research in specific and direct ways in line with a direct use or pipeline model of evidence), conceptual (i.e., more general and indirect form of use in line with a thought enlightenment model of evidence) and political‐symbolic (i.e., use of research knowledge not to inform decision making but to justify a position or action that has already been taken for other reasons in line with a “political model”^10^ uses have been described.^10^, ^11^, ^12^, ^13^ In practice, these uses often overlap. While all three uses of evidence have been operating during the COVID‐19 pandemic,^14^ we argue that the fast pace of decision‐making during a global health emergency has placed much more emphasis on the instrumental use of specific studies as well as bodies of evidence. As a novel feature during the pandemic, evidence has also been used to counter fake news during an unprecedented wave of misinformation.^15^

## 2 OUR APPROACH TO RESPONDING TO THE CHALLENGES

Against this background, there has been a need to review the evidence in an internationally coordinated manner and to develop evidence‐based guidance on PHSM. In response, members of the author team have been engaged with evidence synthesis for PHSM nationally and internationally, notably through the role of the Chair of Public Health and Health Services Research as a WHO Collaborating Centre for Evidence‐Based Public Health and as part of the WHO‐coordinated Evidence Collaboration for COVID‐19 (ECC‐19).^16^, ^17^ Between September 2020 and December 2021, much of our evidence synthesis work was undertaken through the COVID‐19 Evidence Ecosystem (CEOsys) project, funded by the German Federal Ministry of Education and Research, where one work package was exclusively concerned with PHSM. Our efforts to synthesize evidence for decision‐making are embedded in a “knowledge shapes policy” understanding of research‐policy relationships (which may include both instrumental and more conceptual uses of evidence) but also integrates “co‐production” elements (notably with regards to priority topics and questions).^14^, ^18^

**FIGURE 1:**
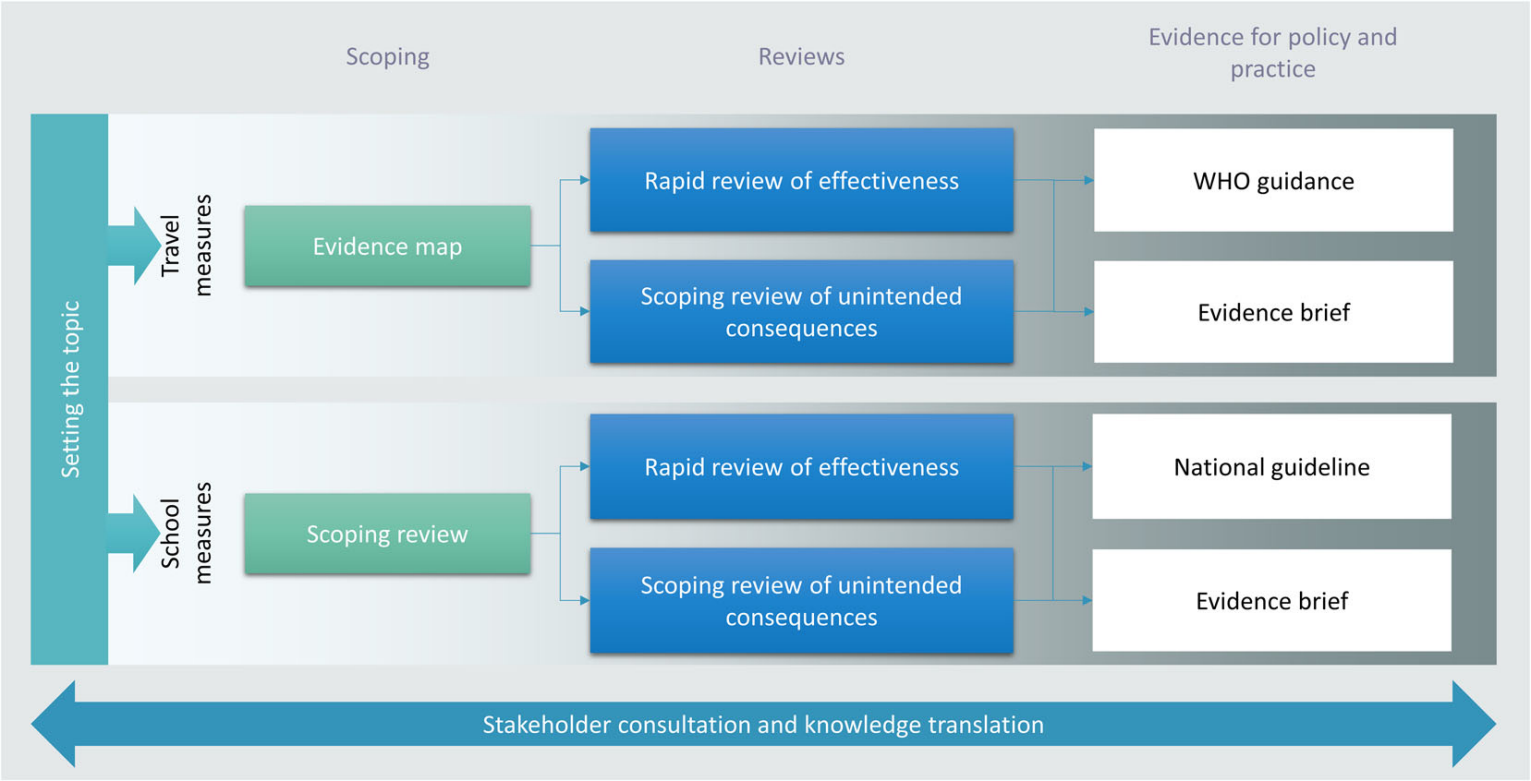
Multi‐component evidence synthesis and knowledge translation strategy [Colour figure can be viewed at wileyonlinelibrary.com]

While we have been focusing on travel measures (a topic set and prioritized by WHO Headquarters) and school measures (a topic identified by us and discussed and agreed upon with the WHO Regional Office for Europe, Cochrane and the CEOsys Public Health Stakeholder Advisory Panel in Germany), this article also integrates lessons learnt with regards to interventions in long‐term care facilities (a topic signaled as important by the CEOsys Public Health Stakeholder Advisory Panel in Germany).^19^ For travel measures and school measures, we pursued a multi‐component evidence synthesis and knowledge translation strategy (Figure 1). This strategy included formally scoping the evidence base, providing specific review products regarding effectiveness and unintended consequences, facilitating the use of findings through evidence briefs^20^ and contributing to evidence‐based guidelines/guidance processes, nationally (for school measures) and/or with WHO (for both sets of measures).^21^, ^22^, ^23^

This article provides an account of our experiences, responses and lessons learnt regarding the above described key challenges when planning and conducting rapid reviews of COVID PHSM over the past 24 months. With this we aim to stimulate further methodological discussions and inform the response of the international review community to future pandemics and other global health emergencies. Related to planning of evidence synthesis, we discuss the value of formal scoping, the importance of utilizing different review types, and considering the evolving nature of the review focus and resulting eligibility. Related to the conduct of evidence synthesis, we outline organizational aspects related to managing such an undertaking on a short timescale, as well as methodological aspects related to rapid reviewing in general and the incorporation of modeling studies in particular.

## 3 PLANNING FOR DIFFERENT TYPES OF REVIEWS

### 3.1 The role and importance of formal scoping

During the COVID‐19 pandemic, with enormous pressure on researchers to collate evidence, investing much time in formal scoping may appear to be a detour. Formal scoping involves consultations with policy and practice stakeholders as end‐users and/or with research stakeholders to ensure that the review to be undertaken is needed and relevant to policy and practice. It also involves conducting a scoping review or an evidence map using systematic and sometimes additional non‐systematic searches across multiple health databases, as well as, where relevant, databases of other disciplines and sectors with the aim to display the volume and characteristics of a body of literature. The development of a conceptual framework or logic model is often helpful, especially when the review is concerned with “complex interventions in complex systems,”^24^, ^25^ as is the case for COVID‐related PHSM. In our experience, the combination of stakeholder consultations and scoping reviews facilitated the development of a meaningful review or package of reviews, and an efficient strategy for moving forward with conducting these. Importantly, formal scoping in our work allowed us to:

- Ensure that the review does not duplicate existing efforts, (*review scope*), thereby avoiding waste of resources.^26^ For example, when we embarked on our Cochrane effectiveness review of school measures we were aware of the published systematic review of the effectiveness of school closures^27^ and, through contact with the authors, knew about planned updates.^28^ In coordination with Cochrane and WHO we therefore deliberately excluded (proactive) school closures from our review scope.
- Identify the needs of users to ensure that the review answers relevant questions (*policy relevance*). For example, during interactions with the WHO Regional Office for Europe and its Technical Advisory Group on Schooling during COVID‐19^29^ the mental health and educational outcomes of children and adolescents were continuously emphasized. In the context of developing evidence‐based and consensus‐based guidelines on school measures during the COVID‐19 pandemic in Germany, where the so‐called WHO‐INTEGRATE decision criteria were applied, it became clear that impacts well beyond health and educational outcomes, for example, the ability for parents of school‐aged children to pursue income‐generating activities, were critical. This prompted us to undertake a scoping review of the unintended consequences of school measures.
- Explore whether evidence exists (*availability of evidence*) and where it may be found (*sources of evidence*). For example, in our evidence map of travel measures, we discovered that very few published or preprint studies in health databases were concerned with outcomes beyond infectious disease control. To assess the broader impacts—positive or negative—of such measures, searches for a broad range of quantitative as well as qualitative studies had to be conducted in non‐health databases, as implemented in our scoping review of unintended consequences.^30^
- Inform decisions regarding the formulation of the review question to enable timely and informative synthesis (*review scope*). Mapping the existing evidence using a broad question helped to delineate the boundaries of the evidence base and highlighted what might be feasible to include in a subsequent evidence synthesis. For example, WHO experts commissioning the travel measures review were initially interested in examining both international and domestic travel measures. However, our scoping identified a very large evidence base, including hundreds of studies addressing a very heterogeneous set of policy questions and measures, which would have been infeasible to synthesize in an informative way in a single review. Based on this, we decided to only consider international (i.e., cross‐border) travel measures for the rapid review of effectiveness (see Appendix S1 for more details on our rapid reviews of travel^31^ and school measures).
- Refine the PICO elements and relevant eligibility criteria and provide an overview of the types of studies available (*nature of evidence*). For example, in our scoping review of school measures,^32^ we discovered that no single randomized controlled trial—individually or cluster‐randomized—had been conducted and that the vast majority of the evidence base consisted of modeling rather than “real world” studies. Had we only considered randomized or quasi‐experimental studies as eligible for inclusion in our Cochrane review of effectiveness, a single quasi‐experimental study would have been included.^33^ Instead, we decided to include modeling studies with multifold implications for team composition and review methods, as discussed in detail in Section 4.3.

In summary, we found that investing time in formal scoping is time well‐spent and that the combination of stakeholder consultations with a scoping review or an evidence map helps respond to all three of the broad pandemic circumstances and associated challenges described above. It is particularly important during a global health emergency such as the COVID‐19 pandemic, when both the health problem and the measures to counter it are novel and rapidly evolving (challenge 1) and when there is no established evidence base to draw upon (challenge 2). Interactions with stakeholders ensure that the review to be conducted is relevant to policy and practice and that insights are produced in a timely manner (challenge 3). Scoping reviews can also help solve many of the challenges frequently encountered during the conduct of standard effectiveness reviews of public health measures. The fact that Cochrane now recognizes scoping reviews as an important and valuable stand‐alone evidence synthesis product and that our scoping review of school measures paved the way as the first‐ever such review to be registered and published in the Cochrane library, pays tribute to the importance of scoping.

### 3.2 Different review types

During the COVID‐19 pandemic, evidence‐informed decision‐making processes—whether highly formalized guideline processes or political processes that deliberately take evidence into account—have asked a range of questions about PHSM: Which measures can effectively reduce SARS‐CoV‐2 transmission and/or alleviate pressure on the health system (effectiveness)? What is the impact of these measures on health beyond infectious disease control and on society at large (unintended consequences)? And, are these measures acceptable and feasible to implement and, if so, how should they be implemented (implementation considerations)? Taken together, there is a need for evidence to provide answers not only regarding what works, but also for whom and under what circumstances.

This raises questions about the most appropriate review type. In our previous evidence synthesis work regarding public health interventions, for example Cochrane reviews of environmental interventions to reduce consumption of sugar‐sweetened beverages^34^ and of interventions to reduce ambient air pollution,^35^ we sought to address all of these questions in a single review of effectiveness, aiming to capture a broad range of outcomes and to extract detailed information on context and implementation aspects. In struggling to define the eligibility criteria for a WHO‐commissioned review on travel measures during COVID‐19 (e.g., international vs. domestic travel measures, transmission‐related outcomes vs. other health and non‐health outcomes; see also further examples in Section 3.3), we proposed that it might be more appropriate to start by mapping the evidence base in a comprehensive manner, conducting systematic searches across databases covering multiple disciplines. Indeed, the resultant evidence map, completed over the course of 10 days, turned out to be of great value in understanding what types of studies were available, which outcomes these assessed and through which databases these might be identified. It made us decide to conduct two separate reviews, a Cochrane rapid review of effectiveness and a scoping review of unintended consequences, and paved the way for the overall multi‐component strategy implemented in CEOsys Public Health. We decided to undertake a scoping review to explore the unintended (and potentially adverse) effects of international travel measures for health and society across multidisciplinary databases, thereby seeking to identify empirically reported unintended consequences in a comprehensive manner. Such a scoping review should normally be followed by a systematic review of unintended consequences undertaking formal evidence synthesis; we did not pursue this, primarily due to the limited and highly heterogeneous evidence base on unintended consequences.

In summary, during a global pandemic—given rapid changes in the health problem and the evidence base even more so than during a normal situation—it is critical to examine the benefits as well as harms of PHSM across different populations and contexts and to explicitly consider implementation aspects. In view of the evolving and highly heterogeneous nature of the primary studies addressing these distinct aspects (challenge 2), it may be most informative to undertake multiple complementary reviews. While different types of reviews have value, their use and related utility for different end‐users vary (challenge 3). For example, an evidence map or scoping review can be completed rapidly but only provides an overview rather than a systematic assessment of the available evidence; in contrast, a review of effectiveness takes longer and is more demanding to conduct but includes risk of bias assessment of included studies and provides more detailed insights regarding the quantitative impacts of measures.

### 3.3 Review focus and eligibility considerations

During the COVID‐19 pandemic, the most pressing questions and related evidence needs regarding PHSM have evolved due to improved knowledge, a changing policy landscape and associated changing priorities among decision‐makers, and a changing context. Travel and school measures have been priority themes from the beginning of the pandemic through to the present day, but levels of interest and the questions asked by decision‐makers and the media have changed over time. Improved knowledge has affected the measures investigated, for example the scientific and (delayed) political recognition of the importance of airborne or aerosol transmission led to a decreasing emphasis on hygiene measures in the school setting. Similarly, the increasing political importance assigned to keeping international borders open placed more emphasis on other measures to ensure safe travel and to limit importation of cases. Dynamic contextual developments with regards to vaccines (i.e., an initial lack of vaccines, followed by the introduction of effective vaccines and varying levels of vaccine roll‐out and uptake around the world) and variants of concern that change the patterns and speed at which the virus moves between individuals and communities, are of particular importance. In parallel, researchers around the world and from many different disciplines have been seeking to respond to these evolving questions by designing and executing studies in record time, sometimes compromising quality by forgoing critical aspects of study design, power and methodologically rigorous execution. All of these aspects play a key role in how a systematic review can be meaningfully conceived—potentially as a living systematic review—and updated over time.

Specific issues that we encountered with regards to making the best possible use of this evolving evidence base while seeking to be both efficient and truthful to the principles of high quality systematic reviews included:

- The use of *direct* versus *indirect evidence*: During the initial stages of the pandemic, no studies on SARS‐CoV‐2 were available. Content knowledge of infectious disease specialists turned out to be essential when we discussed which other infectious diseases might offer relevant insights with regards to travel measures. We considered diseases (i) of viral origin (ii) with a primarily airborne mode of transmission, (iii) being acute with epidemic/pandemic potential, (iv) sharing clinical features with COVID‐19 and (v) for which vaccination was unavailable or unable to contain an outbreak.^36^ Half a year into the pandemic, enough direct evidence on SARS‐CoV‐2 was available to make the consideration of indirect evidence unnecessary in our first update of the review.^31^
- The consideration of *modeling* versus *empirical evidence*: Had we applied normal Cochrane systematic review standards related to study design, we would have produced empty or almost‐empty reviews for both travel measures and school measures, during the early stages of the pandemic. We noted in going from the first version to the second version of our Cochrane review of travel measures that the quality of the studies is gradually improving, with models becoming more sophisticated and better validated, and rather simplistic empirical studies evolving into more informative empirical studies. In the ongoing update toward a third version, we have been encountering a much expanded empirical evidence base comprising time series and other epidemiological study designs.
- Accounting for *dynamic contextual factors*: The effectiveness and feasibility of all public health interventions is inextricably linked with context, and during the COVID‐19 pandemic this has been compounded by the fact that both relatively static and highly dynamic contextual factors are in operation. With regards to travel measures, for example, geographic location (i.e., island vs. landlocked nations), the demography of the population, and socio‐cultural aspects affecting implementation represent important and relatively stable contextual factors; in contrast, levels of transmission in the countries of origin and arrival, and compliance with measures have varied greatly over short time scales, and variants of concern and rising vaccination rates have further complicated the picture. While we sought to extract data and report on a broad range of contextual factors, included studies rarely reported on these in sufficient detail.

In summary, this rapidly evolving health problem (challenge 1) and rapidly evolving evidence base (challenge 2) has made the systematic review process for PHSM to counter COVID‐19 even more complicated than for “standard” public health interventions. It points to the need for regular updates, with the review potentially conceived and conducted as a living review and embedded within a broader “evidence package” on the priority theme from the very beginning. It also suggests that the scope and methodological approach of such a living review might need to change over time, alongside evolving decision‐makers' demands of the evidence and a dynamically changing decision‐making context (challenge 3). This raises many methodological questions, regarding when to update (e.g., on a regular basis, based on a certain number of new studies or when conclusions are likely to change), which aspects to adapt (e.g., adapting the scope and related inclusion criteria in response to improved knowledge and/or pandemic developments) and how to update (e.g., through semi‐automated processes in the context of an evidence ecosystem).

## 4 CONDUCTING THE REVIEW

### 4.1 Organizational aspects when operating on a short timescale

Producing a review on a timescale of a few days or weeks compared to undertaking the same task over many months or even multiple years is associated with a range of organizational challenges. This applies both when the review is requested by a public health institution with an imposed deadline and when the review is researcher‐initiated to meet a real or perceived urgent policy need. The time we took to produce a review and submit a report or manuscript was more affected by “emergency mode” versus “rapid mode” reviewing during different stages of the COVID‐19 pandemic than by the type of review conducted (i.e., evidence map vs. rapid review of effectiveness vs. scoping review of unintended consequences). It ranged from 10 days for our first rapid review in April 2020^37^ to two to 4 weeks for reviews produced in mid‐2020^32^, ^36^ to between 3 and 6 months for reviews produced in late‐2020 and 2021.^31^, ^38^

We undertook our first COVID‐19‐related rapid review with a large review team and a single‐person lead, putting all other research activities on hold. Learning from this experience and over time, our approach to the composition of and roles within our review teams became more sophisticated. Most of our reviews were characterized by a large team (ranging from 14 to 23 authors^38^), consisting of a core team and sub‐teams with specific tasks (e.g., screening, risk of bias assessment, GRADE assessment). More specifically, we instituted a *multi‐person lead* (two first authors, one senior author) that took overall responsibility for developing the protocol, organizing, and managing the review process, ensuring the accuracy of all processes and the validity of findings and writing the review. They were supported by additional members of the *core team*, often the leads of sub‐teams, contributing specific methodological expertise. We ensured that within the review team, *all necessary methodological skills* were present—notably, we recruited several colleagues with different types of modeling expertise. Ensuring *necessary content expertise* was more challenging: while we involved an expert in the management of infectious disease outbreaks in our reviews of travel measures, our reviews on the unintended consequences would have benefited from a more interdisciplinary approach involving legal and political expertise. Being able to undertake complex reviews with large review teams, sometimes with two or three review projects running in parallel, requires forward‐looking and highly flexible recruitment, for example by working together across multiple institutions and by employing team members across different countries and time zones and investing in a range of virtual mechanisms to facilitate onboarding. Capacity‐building on the job is critical: many of our more junior team members moved from a stage of shadowing (i.e., observing what more complicated stages of the review process entail), through a stage of contributing (e.g., being members of the sub‐teams on screening or data extraction) to a stage of leading (i.e., being in charge of a sub‐team or overall review project).

Managing a review project over such a short period of time involves an enormous amount of time and effort as well as skill for coordination and organization. Notably, many tasks that normally occur in sequence (e.g., screening of all records is completed before data extraction commences) take place in parallel. This requires good planning and clear communication, as well as a digital project platform that is easily accessible, easy to use and compliant with data protection regulations. We used a mix of “around the clock” communication within the multi‐person lead team, regular written updates (full review team) and daily (emergency mode) or weekly (rapid mode) meetings (full review team, core team or sub‐teams). To ensure the accuracy of processes, we employed calibration exercises for all key stages of the review process (i.e., title and abstract screening, full‐text screening, data extraction, risk of bias assessment) and kept a list of “rolling questions” to be discussed and resolved during the regular meetings.

In summary, when embarking on a review developed in “emergency mode” or “rapid mode” it is important to be realistic about the required working hours—these are often no fewer than those needed for a review developed in “normal mode” but compressed into a (very) short time period (challenge 3). Importantly, the time taken to peer‐review and publish the review ranged from 8 weeks to 10 months and in several cases was much longer than the time taken to produce the review. The considerable organizational challenges can be met through good planning and management, both for setting up and training the appropriately composed and skilled review team and for organizing and quality controlling the review process.

### 4.2 Methodological aspects with regards to rapid reviewing

Responding to the urgent need for guidance and drawing on ongoing methodological work, Cochrane published interim guidance on rapid review methods.^39^, ^40^ Compared to standard Cochrane systematic reviews, the interim guidance recommends several abridged procedures when conducting a rapid review. In Table 1, we provide an overview of how we implemented and adapted these recommendations in our rapid reviews on travel measures and school measures, differentiating between a rapid review approach in line with Cochrane guidance, adaptations of the rapid review approach as well as a full review approach. In the Appendix S1, we provide a detailed description of all adaptations.

**TABLE 1:** Overview of the differences between rapid review guidance and implementation or adaptation in two rapid reviews of effectiveness of travel measures and school measures

	Cochrane guidance on rapid reviews^40^		Travel measures	School measures
Scoping review	‐		Adaptations of RR approach	Adaptations of RR approach
Research question	*Involve key stakeholders* to set and refine scope		RR approach	RR approach
	Consult with stakeholders throughout the process		Adaptations of RR approach	Adaptations of RR approach
	Develop a *protocol*		RR approach	RR approach
Eligibility criteria	*Define* the population, intervention, comparator and outcomes.		RR approach	RR approach
	Limit the number of interventions and comparators.		RR approach	RR approach
	Limit the number of outcomes		RR approach	FR approach
	Date restrictions		FR approach	FR approach
	Setting restrictions		RR approach	RR approach
	Limit the publication language to English.		Adaptations of RR approach	Adaptations of RR approach
	Include systematic reviews (SRs)		Adaptations of RR approach	Adaptations of RR approach
	Emphasis on higher quality study designs		Adaptations of RR approach	Adaptations of RR approach
Search methods	Involve an information specialist.		RR approach	RR approach
	Limit main database searching		RR approach	RR approach
	*Peer review* of search strategy		RR approach	RR approach
	Limit gray literature and supplemental searching		FR approach	FR approach
	Search *study registries* and scan the *reference lists* of other SRs		RR approach	RR approach
Study selection	Title/abstract screening	Calibration	RR approach	RR approach
		*Double screening* of 20% of studies	FR approach	FR approach
		One author screens remaining abstracts; second author screens excluded abstracts	FR approach	FR approach
	Full text screening	Calibration	RR approach	RR approach
		One author screens included full‐texts; second author screens excluded full‐texts	FR approach	FR approach
Data extraction	Single data extraction and validation by second author		RR approach	FR approach
	Limit data extraction		FR approach	FR approach
	Consider using data from existing SRs		Adaptations of RR approach	Adaptations of RR approach
Risk of bias assessment	Valid risk of bias tool		RR approach	RR approach
	Single risk of bias assessment; validation by second author		RR approach	RR approach
	*Limit risk of bias ratings* to the most important outcomes		FR approach	FR approach
Synthesis	Synthesize evidence narratively.		RR approach	RR approach
	*Meta‐analysis* only if appropriate		RR approach	RR approach
	*Standards* for conducting a *meta‐analysis for an SR* equally apply to an RR.		RR approach	RR approach
	*Grading* of certainty of evidence by a *single author*, *verification* of judgments by second author.		FR approach	FR approach
Other	Protocol approved by Cochrane		RR approach	RR approach
	Protocol published		RR approach	RR approach
	Allow for *post hoc changes* to the protocol		RR approach	RR approach
	Document all post hoc changes; incorporate online SR software		RR approach	RR approach

*Note*: Gray shading in the third and fourth columns indicates where we either adapted the rapid review guidance (light gray) or when we adopted a full review approach (dark gray).

Abbreviations: FR, full review; RR: rapid review.

As shown in Table 1, we adhered to the rapid review guidance issued by Cochrane as much as possible but made adjustments with regards to the eligibility criteria, study selection, risk of bias assessment and synthesis, usually implementing a full systematic review approach rather than introducing further shortcuts. These adjustments are outlined here; those representing a direct consequence of the inclusion of modeling studies are described in further detail in Section 4.3. Limiting the scope of the rapid reviews by imposing very strict *eligibility criteria* was only possible to some degree: both reviews represent reviews with a broad scope covering a range of PHSM, and we imposed few restrictions on the measures, outcomes or settings. Other than a starting date of January 1, 2020, which was implemented for school measures (but not for travel measures) to coincide with the publication of the first COVID‐19‐related studies, no restrictions were imposed on search dates. For *study selection* (i.e., screening), we learnt that taking shortcuts at this stage not only compromised the quality of the review but also created additional problems during later stages of the review process. Therefore, title and abstract as well as full text screening were done in duplicate in both reviews; we employed extensive calibration (e.g., the same 50 studies and the same 10 studies were screened by all authors involved with title and abstract and full‐text screening, respectively, see Appendix S1), a rolling list of questions and regular meetings. Nevertheless, decisions regarding the eligibility of many studies, especially when they differed from more traditional intervention studies (e.g., modeling studies, observational studies) were not straightforward, and discussing their suitability within the core team was important. *Risk of bias assessment* of these types of studies also proved challenging. Understanding their strengths and limitations requires substantial expertise across a range of study types, notably modeling studies, the use of tools not generally used in effectiveness reviews as well as extensive calibration and discussion among the risk of bias sub‐team. These non‐traditional study types often assessed the impact of interventions without providing one single estimate (e.g., where modeling studies explore multiple scenarios, or where observational studies track how an effect develops over time), which made standard quantitative *evidence synthesis* difficult. We had to synthesize the findings narratively and develop an approach to best present the findings in the Summary of Findings tables and the Results section of the review. In doing so, we sought to balance the required level of detail and overly long descriptions, and to avoid too many redundancies in presentation.

In summary, given the complexity of the PHSM under investigation, and of the evidence base (challenges 1 and 2), it was often not sensible to implement the methodological shortcuts suggested by the rapid review guidance and the few shortcuts implemented only yielded very limited time savings. Finding the right balance between, on the one hand, saving time and resources and, on the other hand, producing a high‐quality product useful for researchers and decision‐makers, is challenging, and making sensible decisions will largely depend on the nature and scope of the review. This balance is also likely to depend on whether reviews are conducted in an emergency mode or a rapid mode, as well as on available review team capacity.

### 4.3 Methodological aspects related to modeling studies

Modeling studies have traditionally not been considered in evidence syntheses for health‐related questions. Early in the COVID‐19 pandemic, however, researchers and decision‐makers alike recognized the utility of modeling studies for predicting the impacts of PHSM, in part because of their flexibility and the rapid nature in which they can be conducted. Modeling studies comprise many different classes of models, for example compartmental models, agent‐based models and Bayesian hierarchical models. As described above, for travel measures and school measures, they represented the bulk of the evidence during the first year of the pandemic. Their incorporation into reviews of effectiveness was not without substantial challenges, and required the adaptation of existing methods as well as the development of new methods. These challenges included:

- *Selection*: in defining eligibility criteria for the scoping review, we felt it sufficient to define “travel‐related control measures” as the intervention of interest but determining whether a modeling study appropriately simulates a particular intervention of interest was not straightforward. One type of study we encountered explicitly describes a 90% reduction of incoming flights to mimic closing the borders of a specific country; such studies have clear policy relevance. Another type of study refers to “travel restrictions” in passing and simulates a 10%, 50% and 70% reduction of imported COVID‐19 cases into a large simulated geographical area; such studies may be implicitly relevant, but the link to a concrete policy measure is much less clear. How to operationalize eligibility criteria to distinguish between studies that are more and less policy‐relevant is difficult.
- *Risk of bias assessment*: When we began work on the review of effectiveness of travel measures we searched for an established and validated risk of bias assessment or critical appraisal tool for modeling studies, but were unable to find one. In cooperation with colleagues with modeling expertise, we developed a bespoke tool, which focused on the domains of: model structure, input data, validation, uncertainty and transparency (manuscript in preparation). This tool allowed us to consistently and comprehensively assess all included modeling studies in the travel and school measures reviews and helped us to identify specific patterns, for example, that very few studies assessed the internal and external validity of the respective model. A consistent and comprehensive assessment also enabled us to distinguish between more and less informative modeling studies.
- *Synthesis and managing heterogeneity*: Both the travel and school measures reviews were characterized by vast differences in methodological approaches and scenarios assessed in modeling studies, making meaningful syntheses across studies challenging. Traditionally, evaluations of interventions focus on a single comparison, at most they include a small number of comparisons. In contrast, a single modeling study on alternating school attendance, for example, may investigate multiple options for alternating between different groups, days and weeks; it may also investigate the influence of varying levels of community transmission; additionally, it may investigate the influence of the concurrent school use of masks at varying levels of compliance. This level of complexity across all included studies quickly becomes unwieldy, and raises questions about how to synthesize the evidence and assess heterogeneity. In the effort to summarize these studies, we considered and followed many aspects of the guidance for conducting synthesis without meta‐analysis (SWiM).^41^ This allowed us to provide transparent and structured results. However, unanswered questions remain about how to synthesize and present modeling studies in the most appropriate and informative manner. With regard to the assessment of heterogeneity, the differences between the aspects assessed in included modeling studies were too vast to compare across studies in a meaningful manner. Instead, given the number of interventions and context scenarios covered in single modeling studies, we assessed the impact of heterogeneity on the intervention effect as described within each individual study.
- *Certainty of evidence*: In applying GRADE and as often implemented in reviews of public health interventions, we focused on differences from the null as the meaningful effect threshold in the reviews.^42^ Considering the high disease burden of SARS‐CoV‐2, we viewed any measure allowing for a potential reduction of infections at a population level as important. The use of the bespoke critical appraisal tool, as described above, meant that the “risk of bias” domain could be assessed in a straightforward manner. Assessing other domains, such as imprecision and inconsistency, even with the published guidance on applying GRADE to an evidence base of modeling studies,^43^ was less straightforward. Notably, we had to define rules for operationalization. For example, high‐quality modeling studies may assess a range of different scenarios of different extremes, which could lead to a wide range of potential effects, but it may not mean the effect itself is imprecise; in contrast, a poor‐quality modeling study may not report enough detail to allow for an assessment of imprecision.

In summary, modeling studies have represented an important source of evidence from the beginning of the pandemic until today, yet their incorporation into reviews of effectiveness presents significant challenges (challenge 2). Our experience suggests that authors should take particular care in planning and conducting reviews to ensure that the selection, appraisal, synthesis and GRADE assessment of modeling studies leads to a product that can be used by decision makers; as of yet, validated hands‐on guidance on how to do so is missing. Additionally, it demonstrates the importance of involving researchers with modeling expertise; in the future, further collaboration with such experts could help to ensure that modeling studies are handled in the most appropriate and informative manner in reviews of effectiveness.

## 5 UTILITY OF EVIDENCE SYNTHESIS PRODUCTS: TOWARD A MORE SOLID EVIDENCE BASE ON PUBLIC HEALTH AND SOCIAL MEASURES

Consolidating our experiences with rapid reviewing over the course of the COVID‐19 pandemic, we summarize the lessons that we learnt in planning and conducting rapid reviews of COVID PHSM (see Table 2). Importantly, rapid reviews can feature in different decision‐making processes in a global pandemic. This may involve technical or instrumental applications, such as in guideline development processes to directly inform policy recommendations (e.g., WHO recommendations on travel measures), but may also serve a more symbolic function, when synthesis is used to legitimize or support certain political interests or decisions (e.g., decision processes regarding schools in Germany where an evidence‐based guideline on school measures represented one of many inputs). Stakeholder engagement from an early phase can shape the review product in terms of its scope, relevance, and ultimately, uptake (see also Figure 1) While rapid reviews can be, and often are, expected to be initiated by decision‐makers, researchers may also drive the process by initiating rapid reviews and carefully disseminating their findings.

**TABLE 2:** Lessons learnt regarding rapid reviews on public health and social measures during a global pandemic

Planning for different types of reviews
Conduct *formal scoping* by means of stakeholder consultation and an evidence map or scoping review (Section 3.1) Objective: To obtain an overview of the availability, nature and sources of evidence and to inform the scope of subsequent reviews that are policy‐relevant and feasible.
Undertake *multiple complementary rapid reviews*, making use of the strengths of different review types (Section 3.2) Objective: To examine the benefits as well as harms of PHSM across different populations and contexts and to consider implementation aspects.
Plan reviews of effectiveness as *adaptable living reviews* (Section 3.3) Objective: To respond to evolving evidence needs and an evolving evidence base with adjustments in review scope and review methods

Conducting the review
Ensure *appropriate review team composition and skills* and invest in planning, coordination and communication (Section 4.1) Objective: To ensure smooth and scientifically rigorous processes and efficient use of the substantial working hours compressed into a short period of time
Balance scientific rigor, practical implications of complexity and likely time savings in *deciding on the appropriate methodological shortcuts* in rapid reviewing (Section 4.2) Objective: To ensure the best possible synthesis product given the short timeframe and existing team capacity and resources
Carefully choose, pilot and implement *review methods for modeling studies* (Section 4.3) Objective: To make the best possible use of modeling studies in reviews of effectiveness and to appropriately distinguish between more valid and informative versus less valid and less informative modeling studies

While the COVID‐19 pandemic has resulted in much increased interest in and appreciation of rapid reviews, conducting such reviews during a global pandemic also presents with a range of challenges as, for example, summarized in a scoping review of studies describing methodological challenges of evidence synthesis.^44^ Several groups have described their experiences in the context of the COVID‐19 pandemic, with many similar lessons learnt emerging. These cover different perspectives, such as those of a rapid review team of the National Public Health Institute in Norway,^45^ of a public health network comprising five German professional societies,^46^ of a group of researchers with extensive experience in a broad range of evidence synthesis activities,^47^ of review authors from low and middle‐income countries,^48^ and of researchers involved in a COVID‐19 evidence ecosystem.^49^

Many of the challenges encountered are exacerbated when conducting rapid reviews of PHSM—and our focus on PHSM and our experience from multiple reviews with multiple iterations thus provides novel, concrete and pragmatic insights. Unfortunately, our reviews mostly did not offer clear conclusions for policy and practice, and were characterized by limited confidence in, as well as limited applicability of, the findings. This was partly due to the nature of the available evidence (i.e., mostly modeling and observational studies), which makes it challenging to directly transfer the findings to the “real world”; it was also due to the lack of more nuanced conclusions regarding contextual and implementation issues, which were difficult to examine in the reviews. Based on this experience, we would like to share a few open questions we have been meditating regarding the utility of rapid reviews in a global pandemic and invite further discussion on these:

- *Nature and standards of rapid reviews*: We encountered challenges in adhering to existing guidance on rapid reviewing. This guidance largely addresses reviews conducted in a “rapid mode” over the course of a few months and with only a few abridged procedures; moreover, it might not be possible to know which procedures are amenable to “shortcuts” at the outset of the review. In contrast, rapid reviewing in an “emergency mode,” such as during the first few months of the pandemic, requires reviews to be developed over the course of several days, when adhering to many of the methodological standards set in the existing guidance is not feasible. This raises the question of how to do rapid reviewing under this “emergency mode,” what standards to apply for this, and how to ensure that shortcuts taken do not meaningfully affect quality.
- *Types of evidence and how to make best possible use of these*: The COVID‐19 pandemic has triggered a previously unimaginable growth in scientific studies, many of these being produced rapidly and with questionable scientific rigor.^50^ Another question therefore relates to how different types of evidence—including observational and modeling studies—and publication formats, notably preprint publications, should be negotiated and prioritized for rapid reviewing in a global pandemic. In our reviews, we invested an enormous amount of time and resources to make sense of a large body of modeling studies, seeking to understand the impact of PHSM as predicted in these studies. In light of our limited findings and conclusions, we ponder whether applying the systematic review approach and methods to modeling studies with the aim to “quantitatively” aggregate findings can do justice to modeling studies. Instead, we wonder whether it might not be a more suitable approach to treat evidence synthesis of empirical studies of effectiveness and stand‐alone high‐quality modeling studies as complementary but separate pieces of evidence to inform decision‐making. For example, an evidence synthesis comprising empirical studies could deliver an estimate of the effect of a particular PHSM; this estimate could then be incorporated into a high quality modeling study to explore how context and implementation considerations related to a specific decision may moderate that effect. In general, it would be important to critically reflect on the existing processes of evidence production and reviewing and exploration of alternative approaches to best inform the management of a global health emergency.^51^

In conclusion, we highlight a few suggestions both for primary research and for rapid reviewing and knowledge translation that we consider important for future research on PHSM in a global pandemic and for better pandemic preparedness. Some of these aspects might also apply to other global health emergencies, depending on the time scale over which these originate (e.g., rapid exponential growth as for COVID‐19 vs. slow development as for HIV/AIDS or climate change) and take place (e.g., short‐lived as for seasonal influenza vs. more long‐lived as for COVID‐19).

For primary research, we need to have clearly defined and readily available protocols and standards for conducting scientifically valid and policy‐relevant studies on the effectiveness, unintended consequences and implementation of PHSM in a timely manner. Importantly, these should represent the needs of communities worldwide encompassing multiple perspectives from citizens, practitioners, and policy‐makers. In primary studies conducted to date, findings often have limited applicability because of the lack of detail in reporting on the intervention, implementation, and contextual issues. There is a particular need for standardization of reporting of modeling studies as these are sometimes the only source of evidence in a global pandemic. If systematic reviews are to draw on the evidence from modeling studies, it would be important for these studies to use harmonized methodological approaches to enable meaningful evidence synthesis.

For rapid reviewing, we need to develop realistic guidance and standards tailored to its various types (scoping vs. effectiveness vs. unintended consequences vs. implementation) and modes (“rapid” vs. “emergency”) and critically re‐examine review processes and functions. This includes the role that rapid reviews should play in decision‐making alongside other forms of scientific evidence during a global pandemic or other health emergency, as well as which types of evidence should be considered to enhance utility. The rapid expansion of the evidence base, including both empirical and non‐empirical literature of varying quality, catalyzed by the COVID‐19 pandemic, is likely to continue to challenge review communities in terms of the timely identification of relevant studies.^52^ Looking to the future, it would be important to explore options how this process could be further facilitated, including through the application of advanced digital technologies and automation.^53^, ^54^, ^55^

Decision‐making on PHSM is often complex, especially in emergency situations. It involves consideration not only of evidence on the effects of the measures regarding a narrow set of health outcomes, but of a range of ethical, legal, and broader societal considerations. Expertise of single review teams working within their silos is not sufficient to produce comprehensive and at the same time nuanced synthesis products. This creates a critical need for interdisciplinary collaborations in the practice of comprehensive evidence synthesis efforts, as well as communication and knowledge translation from the early stages of reviewing.

## AUTHOR CONTRIBUTION

EAR had the idea for this article and developed a detailed outline. EAR, JBB, LMP and AM jointly developed the first draft. All authors reviewed different draft versions of the article and approved the final version.

## CONFLICT OF INTEREST

All authors report being part of the COVID‐19 Evidence Ecosystem (CEOsys) project, funded by the German Federal Ministry of Education and Research. E.A.R., J.B., L.M.P. and A.M. are part of the WHO Collaborating Centre for Evidence‐Based Public Health at the LMU Munich. E.A.R. is a methods editor with Cochrane Public Health and founding member of Cochrane Public Health Europe. E.A.R., J.J.M. and A.M. are members of the GRADE Working Group. J.J.M. is the director of Cochrane Germany and the Freiburg GRADE Center, a center of the GRADE Working Group.

## Supporting information


**APPENDIX S1** Supporting Information

## Data Availability

Data sharing is not applicable to this article as no datasets were generated or analysed during the current study.
