# Supplementary material for: Lessons learnt: Undertaking rapid reviews on public health and social measures during a global pandemic
Source: Res Synth Methods. 2022 Jul 31;13(5):558–72. doi: 10.1002/jrsm.1580 (PMC9349463; doi:10.1002/jrsm.1580)
Supplement: Supplementary file 1 — APPENDIX S1 Supporting Information [file JRSM-13-558-s001.pdf]

Undertaking rapid reviews on public health and social measures during a global pandemic

Rehfuess EA, Burns JB, Pfadenhauer LM, Krishnaratne S, Littlecott H, Meerpohl JJ, Movsisyan A

SUPPORTING INFORMATION

Table S1: Review methods recommended in Cochrane guidance and employed in rapid reviews of effectiveness of travel measures and school measures

This table provides an overview of how we implemented and adapted Cochrane guidance on rapid reviews (1) in our rapid reviews of the effectiveness of travel measures and school measures. We differentiate between a rapid review approach in line with Cochrane guidance (RR approach), adaptations of the rapid review approach (adaptations of RR approach) as well as a full review approach (FR approach). Grey shading of table cells indicates where the approach taken in our rapid reviews differed from Cochrane guidance on rapid reviews.

	Cochrane guidance on rapid reviews	Rapid review of travel measures	Rapid review of school measures
Scoping review	-	Adaptations of RR approach:<br>Evidence map conducted prior to full review, alongside stakeholder engagement.	Adaptations of RR approach:<br>Cochrane scoping review conducted prior to full review, alongside stakeholder engagement.
Research question	Involve key stakeholders (e.g., review users such as consumers, health professionals, policy-makers, decision-makers) to set and refine the review question, eligibility criteria, and the outcomes of interest.	RR approach:<br>The research team was approached by WHO with a broad topic of interest. The review question and scope were defined through discussions with the WHO and content experts.	RR approach:<br>The topic was identified by the review team and discussed and agreed upon with the WHO Regional Office for Europe and the CEOsys Public Health Stakeholder Advisory Panel in Germany.
	Consult with stakeholders throughout the process to ensure the research question is fit for purpose, and regarding any ad-hoc changes that may occur as the review progresses.	Adaptations of RR approach:<br>We did not consult stakeholders throughout the process.	Adaptations of RR approach:<br>We did not consult stakeholders throughout the process.
	Develop a protocol that includes review questions, PICOS, and inclusion and exclusion criteria.	RR approach:<br>The protocol, registered with the Open Science Framework, was developed detailing the review	RR approach:<br>The protocol, registered with the Open Science Framework, was developed detailing the review

	Cochrane guidance on rapid reviews	Rapid review of travel measures	Rapid review of school measures
		question, PICOS-related eligibility criteria and all relevant methods.	question, PICOS-related eligibility criteria and all relevant methods.
Eligibility criteria	Clearly define the population, intervention, comparator and outcomes.	RR approach:<br>The protocol, informed by the previously conducted evidence map, clearly defined the population, intervention, comparator and outcomes.	RR approach:<br>The protocol, informed by the previously conducted Cochrane scoping review, clearly defined the population, intervention, comparator and outcomes.
	Limit the number of interventions and comparators.	RR approach:<br>A broad range of travel measures were considered. These were, however, limited to those that cross national borders compared to no measures, complete or partial relaxation of the measures, or an alternative travel measure. Measures implemented during travel were not considered (e.g. mask wearing, ventilation).	RR approach:<br>A broad range of school measures were considered. Intervention and comparators were limited to studies that assess measures implemented in the school setting to safely reopen schools and/or keep schools open during the COVID-19 pandemic, i.e. school closures were not addressed.
	Limit the number of outcomes, with a focus on those most important for decision-making.	RR approach:<br>Primary outcomes were limited to infectious disease-related outcomes and organized in three main categories: cases avoided, cases detected and shift in epidemic development.	FR approach:<br>Primary outcomes were not limited but organized in four broad categories: transmission-related outcomes, healthcare utilization outcomes, other health outcomes, societal, economic, and ecological outcomes.
	Consider date restrictions with a clinical or methodological justification.	FR approach:<br>No restrictions: electronic databases were searched from the earliest date available to the date of the searches (26 June 2020 for first review, 13 November 2020 for the update).	FR approach:<br>No restrictions: electronic databases were searched from 1 January 2020 to the date of the searches (9 December 2020).
	Setting restrictions are appropriate with justification provided.	RR approach:<br>The setting was limited to international travel.	RR approach:<br>The setting was limited to school settings (i.e. no childcare settings for children <5 years of age, no university/college setting).
	Limit the publication language to	Adaptations of RR approach:	Adaptations of RR approach:

	Cochrane guidance on rapid reviews	Rapid review of travel measures	Rapid review of school measures
	English; add other languages only if justified.	Searches were conducted in English. Publications in any of the following languages were considered: Armenian, English, French, German, Italian, Russian, and Spanish.	Searches were conducted in English. Publications in any of the following languages were considered: Armenian, English, French, German, Italian, Russian, and Spanish.
	Systematic reviews (SRs) should be considered a relevant study design for inclusion.	Adaptations of RR approach:<br>SRs were not included as a relevant study design.	Adaptations of RR approach:<br>SRs were not included as a relevant study design.
	Place emphasis on higher quality study designs (e.g., SRs or RCTs); consider a stepwise approach to study design inclusion.	Adaptations of RR approach:<br>All studies providing a quantitative measure of impact were considered (i.e. experimental, observational and modelling).	Adaptations of RR approach:<br>All studies providing a quantitative measure of impact were considered (i.e. experimental, observational and modelling).
Search methods	Involve an information specialist.	RR approach:<br>The review search strategy was designed and run by a Cochrane information specialist.	RR approach:<br>The review search strategy was designed and run by a Cochrane information specialist.
	Limit main database searching to CENTRAL, MEDLINE (e.g., via PubMed), and Embase (if available). Searching of specialized databases (e.g., PsycInfo and CINAHL) is recommended for certain topics but should be restricted to one or two additional sources, or omitted if time and resources are limited.	RR approach:<br>Database searches included: Ovid MEDLINE, Ovid Embase, Cochrane COVID-19 Study Register, WHO 'global literature on coronavirus disease', CDC COVID-19 Research Database (the latter for the first review only, the content of which was later integrated with that of and accessed through the WHO 'global literature on coronavirus disease'.	RR approach:<br>Databases searched included: Ovid MEDLINE, Ovid Embase, Cochrane Central Register of Controlled Trials (CENTRAL), Educational Resources Information Center (ERIC), Cochrane COVID-19 Register, WHO 'global literature on coronavirus disease'.
	Consider peer review of at least one search strategy (e.g., MEDLINE).	RR approach:<br>All search strategies were peer-reviewed by a Cochrane information specialist.	RR approach:<br>All search strategies were peer-reviewed by a Cochrane information specialist.
	Limit grey literature and supplemental searching	FR approach:<br>Searches were conducted on preprint servers.	FR approach:<br>Searches were conducted on preprint servers and Google.
	If justified, search study registries and scan the reference lists of other SRs, or	RR approach:<br>Backward searching was conducted on previously	RR approach:<br>Forward and backward searching was conducted

	Cochrane guidance on rapid reviews	Rapid review of travel measures	Rapid review of school measures
	included studies after screening of the abstracts and full-texts.	published systematic reviews.	on previously published systematic reviews, guidelines and included studies.
Study selection	Title and abstract screening: Using a standardized title and abstract form, conduct a pilot exercise using the same 30 or 50 abstracts for the entire screening team to calibrate and test the review form.<br><br>Use two authors for dual screen of at least 20% (ideally more) of abstracts, with conflict resolution.	RR approach:<br>To pilot and harmonize the title and abstract screening process, the same 50 studies were screened by all authors involved in screening using a standardized screening form.	RR approach:<br>To pilot and harmonize the title and abstract screening process, the same 50 studies were screened by all authors involved in screening using a standardized screening form.
	Title and abstract screening: Use one author to screen the remaining abstracts and a second author to screen all excluded abstracts, and if needed resolve conflicts.	FR approach:<br>Two authors screened all remaining titles and abstracts in duplicate, excluding only those studies which were clearly irrelevant; studies that were marked as unclear were moved forward to the next stage.<br>Any unclear cases were discussed with a third author from the core team and/or within the screening sub-team. Moreover, a rolling question sheet collected all arising questions which were discussed in regular team meetings.	FR approach:<br>Two authors screened all remaining titles and abstracts in duplicate, excluding only those studies which were clearly irrelevant; studies that were marked as unclear were moved forward to the next stage.<br>Any unclear cases were discussed with a third author and/or within the screening sub-team. Moreover, a rolling question sheet collected all arising questions which were discussed in regular team meetings.
	Full-text screening: Using a standardized full-text form, conduct a pilot exercise using the same 5-10 full-text articles for the entire screening team to calibrate, and test the review form.	RR approach:<br>To pilot and harmonize the full-text screening process, the same 10 studies were screened by all authors involved with full-text screening using a standardized screening form.	RR approach:<br>To pilot and harmonize the full-text screening process, the same 10 studies were screened by all authors involved with full-text screening using a standardized screening form.
	Full-text screening: Use one author to screen all included full-text articles and	FR approach:<br>Two authors screened the remaining full texts in	FR approach:<br>Two authors screened the remaining full texts in

	a second author to screen all excluded full-text articles.	duplicate. Any unclear cases were discussed with a third author from the core team and/or within the screening sub-team. Moreover, a rolling question sheet collected all arising questions which were discussed in team meetings.	duplicate. Any unclear cases were discussed with a third author and/or within the screening sub-team. Moreover, a rolling question sheet collected all arising questions which were discussed in team meetings.
Data extraction	Use a single author to extract data using a piloted form. Use a second author to check for correctness and completeness of extracted data.	RR approach:<br>The data extraction form was piloted on three studies with different intervention types.<br><br>One author extracted the data from the included studies, and at least one further author checked those for completeness and correctness.	FR approach:<br>The data extraction form was piloted on five studies with different intervention types.<br><br>Two authors independently extracted study characteristics and data from all included studies using a data extraction form.
	Limit data extraction to a minimal set of required data items.	FR approach:<br>Data were extracted in the following main categories in a relatively detailed manner: study information, study type, PICO elements, setting, context. For the first review information on the pathogen/ disease was also extracted, the update was limited to SARS-CoV-2/COVID-19.	FR approach:<br>Data were extracted in the following main categories in a relatively detailed manner: study information, study type, PICO elements, setting, context and implementation.
	Consider using data from existing SRs to reduce time spent on data extraction.	Adaptations of RR approach:<br>We did not use data from existing SRs.	Adaptations of RR approach:<br>We did not use data from existing SRs.
Risk of bias assessment	Use a valid risk of bias tool, if available, for the included study designs.	RR approach:<br>Multiple tools were used in assessing the risk of bias or quality of studies, including an established tool for observational studies concerned with screening (QUADAS-2) and a bespoke tool developed by the team for modelling studies.	RR approach:<br>Multiple tools were used in assessing the risk of bias or quality of studies, including established tools for quasi-experimental studies (ROBINS-I) and observational studies concerned with screening (QUADAS-2) and a bespoke tool developed by the team for modelling studies.
	Use a single author to rate risk of bias, with full verification of all judgments (and support statements) by a second author.	RR approach:<br>One author rated the risk of bias or quality of each included study, and a second author checked the judgements. Any conflicts,	RR approach:<br>One author rated the risk of bias or quality of each included study, and a second author checked the judgements. Any conflicts,

		questions, or uncertainties were discussed between these authors and/or among the risk of bias sub-team.	questions, or uncertainties were discussed between these authors and/or among the risk of bias sub-team.
	Limit risk of bias ratings to the most important outcomes, with a focus on those most important for decision-making.	FR approach:<br>Risk of bias or quality ratings were applied to all studies providing outcomes in the three broad pre-specified categories of primary outcomes.	FR approach:<br>Risk of bias or quality ratings were applied to all studies providing outcomes in the four pre-specified categories of primary outcomes.
Synthesis	Synthesize evidence narratively.	RR approach:<br>Meta-analysis was not possible due to the very heterogeneous evidence base identified.<br>The findings were synthesized narratively, stratified by intervention type and outcome, and guided by SWiM guidance. A phased approach was used for this: (i) study-by-study tables were created describing the direction of intervention effects; (ii) summary of findings and abstracted narratives were developed for each intervention category and primary outcome across the contributing studies; (iii) the overall direction of effect was determined for each intervention-outcome pair.<br><br>Findings from empirical and modelling studies were synthesized and presented separately. The synthesis was done by two core team authors working collaboratively. All uncertainties in the process were discussed and resolved with the third core team author.<br><br>All data presented in the tables, text and graphics were further checked by a third author.	RR approach:<br>Meta-analysis was not possible due to the very heterogeneous evidence base identified. The findings were synthesized narratively, stratified by intervention type and outcome. A phased approach was used for this: (i) study-by-study tables were created describing the direction of intervention effects; (ii) summary of findings and abstracted narratives were developed for each intervention category and primary outcome across the contributing studies; (iii) the overall direction of effect was determined for each intervention-outcome pair.<br><br>Findings from empirical and modelling studies were synthesized and presented separately. The synthesis was done by one core team author and double-checked by a second core team author.<br><br>All data presented in the tables, text and graphics were double-checked by a third author.
	Consider a meta-analysis only if appropriate (i.e., studies are similar	RR approach:<br>Studies were too heterogeneous to conduct a	RR approach:<br>Studies were too heterogeneous to conduct a

	enough to pool).	meta-analysis.	meta-analysis.
	Standards for conducting a meta-analysis for an SR equally apply to an RR.	RR approach:<br>We did not conduct a meta-analysis.	RR approach:<br>We did not conduct a meta-analysis.
	Use a single author to grade the certainty of evidence, with verification of all judgments (and footnoted rationales) by a second author.	FR approach:<br>For GRADE, one author suggested initial certainty of evidence ratings, which were further deliberated in a sub-team responsible for this task, and a joint decision was made.	FR approach:<br>For GRADE, the assessment was undertaken in the core team, by at least two authors, and a joint decision was made.
Other	RRs should be preceded by a protocol submitted to and approved by Cochrane	RR approach:<br>The protocol was peer reviewed and approved by Cochrane prior to the conduct of the review.<br><br>The review was updated once since the publication of the original version, and a second update will commence soon.	RR approach:<br>The protocol was peer reviewed and approved by Cochrane prior to the conduct of the review.<br><br>As more than 6 months passed between the publication of the study protocol and submission of the full review to Cochrane, a top-up search of the Cochrane Study Register was conducted 7 months after the original search to identify (but not extract data or synthesize data from) additional studies.
	Protocol should be published (e.g., PROSPERO or Open Science Framework)	RR approach:<br>The protocol was registered with the Open Science Framework and appended to the main review.	RR approach:<br>The protocol was registered with the Open Science Framework.
	Allow for post hoc changes to the protocol (eligibility criteria etc.) as part of an efficient and iterative process	RR approach:<br>We allowed for post hoc changes.	RR approach:<br>We allowed for post hoc changes.
	Document all post hoc changes; and incorporate use of online SR software (e.g., Covidence, DistillerSR, and EPPI-Review author) to streamline the process.	RR approach:<br>All post hoc changes to the protocol were highlighted and documented in the review publication. There were no major post hoc changes; minor post hoc changes referred to the title, criteria for considering studies in the	RR approach:<br>All post hoc changes to the protocol were highlighted and documented in the review publication. There were no major post hoc changes; minor post hoc changes referred to restructuring the intervention categories,

		review, data extraction and management, assessment of risk of bias, data synthesis, investigation of heterogeneity and sensitivity analyses.	assessment of risk of bias, and data synthesis.

Reference:

1. Garritty C, Gartlehner G, Kamel C, King V, Nussbaumer-Streit B, Stevens A. Interim guidance from the Cochrane rapid reviews methods Group. 2020. Cochrane rapid reviews[Google Scholar]. 2020.
